# Retinal microvascular parameters are not associated with reduced renal function in a study of individuals with type 2 diabetes

**DOI:** 10.1038/s41598-018-22360-3

**Published:** 2018-03-02

**Authors:** Gareth J. McKay, Euan N. Paterson, Alexander P. Maxwell, Christopher C. Cardwell, Ruixuan Wang, Stephen Hogg, Thomas J. MacGillivray, Emanuele Trucco, Alexander S. Doney

**Affiliations:** 1 Centre for Public Health, Queen’s University Belfast, Belfast, Northern Ireland; 2 VAMPIRE project, Computer Vision and Image Processing Group, School of Science and Engineering (Computing), University of Dundee, Dundee, United Kingdom; 3 VAMPIRE project, Centre for Clinical Brain Sciences, University of Edinburgh, Edinburgh, United Kingdom; 4 Ninewells Hospital and Medical School, University of Dundee, Dundee, United Kingdom

## Abstract

The eye provides an opportunistic “window” to view the microcirculation. There is published evidence of an association between retinal microvascular calibre and renal function measured by estimated glomerular filtration rate (eGFR) in individuals with diabetes mellitus. Beyond vascular calibre, few studies have considered other microvascular geometrical features. Here we report novel null findings for measures of vascular spread (vessel fractal dimension), tortuosity, and branching patterns and their relationship with renal function in type 2 diabetes over a mean of 3 years. We performed a nested case-control comparison of multiple retinal vascular parameters between individuals with type 2 diabetes and stable (non-progressors) versus declining (progressors) eGFR across two time points within a subset of 1072 participants from the GoDARTS study cohort. Retinal microvascular were measured using VAMPIRE 3.1 software. In unadjusted analyses and following adjustment for age, gender, systolic blood pressure, HbA_1C_, and diabetic retinopathy, no associations between baseline retinal vascular parameters and risk of eGFR progression were observed. Cross-sectional analysis of follow-up data showed a significant association between retinal arteriolar diameter and eGFR, but this was not maintained following adjustment. These findings are consistent with a lack of predictive capacity for progressive loss of renal function in type 2 diabetes.

## Introduction

Type 2 diabetes mellitus is a disease characterised by micro- and macrovascular complications in 30–50% of people with the condition^1^ and is the commonest cause of end-stage renal disease (ESRD), accounting for approximately 45% of incident and 38% of prevalent ESRD in the United States^2^. High rates of incident chronic kidney disease (CKD) have been reported in both type 1 and type 2 diabetes based on established renal function cut-offs (estimated glomerular filtration rate (eGFR) <60 ml/min/1.73 m^2^), with up to 29% of those with newly diagnosed type 2 diabetes likely to develop CKD over 15 years of follow-up^3^, with similar rates also reported in type 1 diabetes^4,5^.

Pathological microvascular changes that manifest themselves in diabetes are important contributory mechanisms leading to increased prevalence of CKD in those with the condition^6–8^. The retina enables accessible visualisation of tissue vascularisation and quantitative evaluation of retinal microvascular parameters (RVP) and specific retinal microvascular changes have been associated with a range of vascular-related conditions, including hypertension^9^, coronary heart disease^10^, stroke^11^ and diabetes^12^. Retinal microvascular parameters have also been suggested to reflect systemic microvascular damage resulting from renal dysfunction^13^, and in population-based studies, reduced eGFR has been associated with both narrower retinal arteriolar^14–21^ and venular calibre^14,16,22,23^, but to date the evidence has been limited and the findings reported, inconsistent.

A wide range of perturbed metabolic pathways are associated with vascular injury in diabetes. Advanced glycation end products are formed, microRNA profiles become attenuated, nitric oxide bioavailability is reduced, oxidative damage is increased and there is a proliferation of inflammatory mediators such as interleukin-6 (IL-6) and c-reactive protein (CRP)^8,24^. Several of these biochemical changes are associated with alterations in retinal vascular calibre, e.g. IL-6 and CRP^25,26^, serum glucose^26^, and nitric oxide inhibition^27^. CKD has also been associated with vascular changes linked to hypertension, dyslipidaemia, endothelial dysfunction, accelerated atherosclerosis, inflammation and abnormal bone mineral metabolism^28,29^. While calcification may influence vascular morphology, hypertension has established associations with narrower retinal arteriolar calibre^30^. The combined vascular effects of type 2 diabetes and renal impairment may correlate with earlier retinal changes which, if identified during routine screening, may enable detection and stratification of those at increased risk of progressive CKD, facilitating earlier clinical intervention to slow CKD progression.

The complexity of the retinal vasculature can be estimated by its fractal dimension. Lower fractal dimension has been reported in those with CKD^18,23^ in contrast to increased fractal dimension in type 2 diabetes^31^. Given the divergent effects of type 2 diabetes and CKD on retinal microvascular fractal dimension, it is unclear if this parameter is associated with diabetes, CKD or both. Increased retinal vascular tortuosity has also been reported in association with longer diabetes duration^32^ but evidence on the impact of change in renal function and retinal vascular tortuosity in diabetes is scarce. The aim of this study therefore was to examine the prognostic potential of RVP to predict eGFR decline in a large prospective cohort of type 2 diabetes.

## Results

Overall the sample population had a mean age of 63.0 years (standard deviation (SD) = 7.6) and 49% were female. Mean follow-up time was 3.01 years (SD = 0.35). The study sample had a mean glycated haemoglobin (HbA_1_c) of 7.41% (SD = 1.39), and a mean systolic blood pressure (SBP) of 138 mmHg (SD 13). A total of 570 participants met the group 1 definition and were designated non-progressors, 335 participants met the group 2 criteria and were designated progressors. There were no significant differences in blood pressure, HbA_1_c or diabetic retinopathy status between progressors and non-progressors at baseline. Baseline eGFR was significantly higher in progressors compared to non-progressors (98.6 ml/min/1.73 m^2^, SD = 21.3 vs. 91.3 ml/min/1.73 m^2^, SD = 14.3, p < 0.001) (Table 1).

**Table 1:** Baseline sample characteristics.

Baseline Variables	Sample n = 1068	Progressors n = 335	Non-progressors n = 570	p
Age, yrs (SD)	63.0 (7.6)	62.5 (7.7)	63.1 (7.8)	0.21
Gender, female (%)	521 (49)	168 (50)	281 (49)	0.81
eGFR, ml/min/1.73 m^2^ (SD)	94.0 (17.2)	98.6 (21.3)	91.3 (14.3)	<0.001
SBP, mmHg (SD)	138 (13)	139 (14)	137 (13)	0.08
DBP, mmHg (SD)	77 (8)	76 (9)	77(8)	0.63
HbA_1C_, % (SD); mmol/mol	7.41 (1.38); 57.5	7.51 (1.36); 58.6	7.40 (1.41); 57.4	0.25
Diabetic retinopathy present, n (%)	244 (23)	82 (25)	118 (21)	0.19
Mean follow-up period, yrs (SD)	3.01 (0.35)	3.02 (0.35)	3.02 (0.34)	0.98

Yrs: years; eGFR: estimated glomerular filtration rate (calculated using the CKD-EPI equation); SBP: systolic blood pressure; DBP: diastolic blood pressure; HbA_1_c: glycated hemoglobin; SD: standard deviation. P values were calculated by independent sample t and chi squared tests for comparisons between progressors and non-progressors.

Over the mean follow up time of 3.01 yrs, mean change in eGFR was −27.73 ml/min/1.73 m^2^ (SD = 14.31 ml/min/1.73 m^2^) in progressors versus +2.74 ml/min/1.73 m^2^ (SD = 10.85 ml/min/1.73 m^2^) in non-progressors, p < 0.001. SBP and diastolic blood pressure (DBP) fell in both groups, but decreased significantly more in progressors than in non-progressors (SBP decreased by 2.49 mmHg (16.26) and 0.23 mmHg (12.94) for progressors and non-progressors respectively, p = 0.04; DBP decreased by 2.61 mmHg (9.36) and 1.15 mmHg (7.69) respectively in progressors and non-progressors, p = 0.02). There was no significant change in HbA_1C_ in both groups between time-points (+0.13% (1.42), and +0.04% (1.37) for progressors and non-progressors respectively, p = 0.41). In both groups a non-significant decrease in arteriolar and venular calibre of approximately 1% was observed between time-points but there was no significant difference in the rate of vascular narrowing between groups. No significant differences in the other parameters measured (fractal dimension, tortuosity, or number of first branches) were detected between progressors and non-progressors (Table 2).

**Table 2:** Between group comparisons for progressors and non-progressors. eGFR: estimated glomerular filtration rate (calculated using the CKD-EPI equation); SD: standard deviation. P values were calculated by independent sample t test. ^a^Tortuosity variables were log transformed before to produce normal distribution.

Variables	Progressors n = 335 Mean change (SD)	Non-progressors n = 570 Mean change (SD)	p
SBP, mmHg	−2.49 (16.26)	−0.23 (12.94)	0.04
DBP, mmHg	−2.61 (9.36)	−1.15 (7.69)	0.02
HbA_1C_, % (SD); mmol/mol	0.13 (1.42); 1.4	0.04 (1.37); 0.4	0.38
eGFR, ml/min/1.73 m^2^	−27.73 (14.31)	2.74 (10.85)	<0.001
Calibre			
Central retinal arteriolar equivalent	−0.46 (2.41)	−0.52 (2.55)	0.72
Central retinal venular equivalent	−0.57 (2.94)	−0.56 (3.41)	0.95
Arteriovenous ratio	4.7 × 10^−5^ (5.0 × 10^−2^)	−1.9 × 10^−3^ (5.5 × 10^–2^)	0.60
No. of first branches in zone C			
Arteriolar	0.01 (1.15)	−0.13 (1.19)	0.09
Venular	−0.03 (1.03)	−0.09 (0.97)	0.32
Fractal dimension			
Arteriolar	−5.5 × 10^−3^ (0.05)	−9.1 × 10^−3^ (0.06)	0.36
Venular	−6.3 × 10^−3^ (0.05)	−9.1 × 10^−3^ (0.05)	0.45
Tortuosity			
^a^Arteriolar	−1.1 × 10^−2^ (0.25)	3.3 × 10^−3^ (0.27)	0.44
^a^Venular	4.5 × 10^−2^ (0.31)	4.0 × 10^−2^ (0.29)	0.81

In unadjusted and adjusted logistic regression models controlling for age, sex, SBP, and HbA_1_c, none of the baseline RVP were significantly associated with greater odds of being a progressor. For instance, per unit increase in central retinal arteriolar calibre (CRAE) the odds of being a progressor were multiplied by 1.02 (OR = 1.02, 95% CI = 0.97, 1.08) in the adjusted analysis but this was not statistically significant (p = 0.46). Similarly, per unit increase in central retinal venular calibre (CRVE), the odds of being a progressor were multiplied by 1.03 (OR = 1.03, 95% CI = 0.99, 1.07) in the adjusted analysis but this was also not statistically significant (p = 0.18). Further adjustment for diabetic retinopathy and fellow vessel calibre also failed to identify any significant associations between RVP and likelihood of decline in eGFR. No associations were identified between baseline RVP and odds of being a progressor in this type 2 diabetes cohort (Table 3).

**Table 3:** Logistic regression models testing associations between baseline retinal vessel parameters and decline in renal function between progressors (cases) and non-progressors (controls). Retinal microvascular parameter and progression of renal functional decline (progressors versus non-progressors) adjusted for age, gender, baseline systolic blood pressure, and baseline HbA_1c_. OR: Odds ratio. 95% CI: 95% confidence interval. ^a^Tortuosity values were multiplied by 1000 before inclusion in logistic regression models to produce meaningful values.

Retinal microvascular parameter (per unit increase)	Unadjusted OR (95% CI)	P	Adjusted OR (95% CI);	P
Calibre				
Central retinal arteriolar equivalent	1.01 (0.96, 1.07)	0.40	1.02 (0.97, 1.08)	0.46
Central retinal venular equivalent	1.03 (0.99, 1.07)	0.16	1.03 (0.99, 1.07)	0.18
Arteriovenous ratio	0.39 (0.05, 3.38)	0.39	0.57 (0.06, 5.47)	0.62
Fractal dimension				
Arteriolar	0.82 (0.08, 8.11)	0.86	0.89 (0.08, 9.76)	0.93
Venular	1.45 (0.14, 14.7)	0.75	1.12 (0.10, 13.0)	0.93
No. of First branches in zone C				
Arteriolar	0.95 (0.85, 1.07)	0.40	0.96 (0.85, 1.07)	0.46
Venular	0.90 (0.80, 1.02)	0.10	0.91 (0.80, 1.03)	0.13
Tortuosity				
^a^Arteriolar	0.68 (0.22, 2.17)	0.68	0.79 (0.24, 2.60)	0.70
^a^Venular	6.88 (0.68, 69.7)	0.10	6.61 (0.63, 69.8)	0.12

Lower CRAE at follow-up was significantly associated with follow-up eGFR in unadjusted linear regression analysis (β = −0.47, 95% CI = −0.87, −0.07, p = 0.02), with CRVE (β = −0.30, 95% CI = −0.60, 0.00, p = 0.05) and arteriolar fractal dimension (β = −18.41, 95% CI = −36.92, 0.10, p = 0.05) approaching statistical significance. After adjustment for age, gender, follow-up SBP and HbA1c, the associations between RVP and eGFR at follow-up were no longer statistically significant (Table 4). Associations were not materially altered following additional adjustment for diabetic retinopathy or fellow vessel calibre (data not shown).

**Table 4:** Linear regression models testing cross-sectional associations between follow-up eGFR and follow-up RVP.

Retinal microvascular parameter (per unit increase)	Unadjusted β eGFR (95% CI)	p	Adjusted β eGFR (95% CI)	p
Calibre				
Central retinal arteriolar equivalent	−0.47 (−0.87, −0.07)	0.02	−0.38 (−0.80, 0.05)	0.08
Central retinal venular equivalent	−0.30 (−0.60, 0.00)	0.05	−0.27 (−0.58, 0.05)	0.10
Arteriovenous ratio	−3.32 (−21.81, 15.16)	0.72	−0.52 (−19.64, 18.60)	0.96
Fractal dimension				
Arteriolar	−18.41 (−36.92, 0.10)	0.05	−17.64 (−36.71, 1.44)	0.07
Venular	−3.74 (−22.79, 15.31)	0.70	−3.46 (−23.36, 16.43)	0.73
No. of First branches in zone C				
Arteriolar	−0.67 (−1.63, 0.30)	0.17	−0.50 (−1.50, 0.49)	0.32
Venular	0.66 (−0.43, 1.75)	0.24	0.82 (−0.31, 1.95)	0.15
Tortuosity				
^a^Arteriolar	−0.01 (−2.66, 2.65)	1.00	−0.01 (−2.75, 2.73)	0.99
^a^Venular	−3.20 (−6.73, 0.32)	0.08	−2.22 (−5.86, 1.43)	0.23

Follow-up eGFR and follow-up RVP, adjusted for age, gender, systolic blood pressure at follow-up, and HbA_1c_ at follow-up. 95% CI: 95% confidence interval. ^a^Tortuosity variables were log transformed before linear regression to produce normal distribution.

## Discussion

Routine investigation of retinal biomarkers has improved through advances in digital imaging systems and routine eye screening programmes, wider availability through high street opticians and software improvements capable of quantifying multiple RVP with improved accuracy.

In this longitudinal, case-control study of individuals with type 2 diabetes, the prognostic value of a wide range RVP for the identification of those at increased risk of eGFR decline was considered. No association between baseline RVP and change in eGFR between two time points approximately 3 years apart was found, suggesting a lack of potential for RVP as predictive biomarkers of eGFR decline in this cohort of type 2 diabetes over the limited time-period considered.

Several studies have previously reported associations between renal disease and RVP in both type 1 and type 2 diabetes. Cross-sectional studies in type 1 diabetes found associations between narrower retinal arterioles and prevalent diabetic nephropathy (DN)^33,34^. In contrast, prospective studies of type 1 diabetes have reported wider venular calibre in association with albuminuria^35,36^ and renal insufficiency^35^ over 16 years of follow-up, while narrower retinal arterioles^36^ and sparser vasculature^37^ have also been reported in association with proteinuria. These previous findings in type 1 diabetes contrast to the present study which found no significant associations with eGFR decline in type 2 diabetes. Reductions in eGFR can occur in the presence or absence of albuminuria^38^ and may have entirely independent associations with retinal vascular morphology. Population differences between type 1 and type 2 diabetes are also worthy of further consideration. Age is a well-established confounder of RVP and type 1 diabetes populations are typically younger. The population of the Danish Cohort of Pediatric Diabetes^36^ had a mean age of 21 years, far younger than the 63 year average age of the population of this study. Age-related variation leads to greater “noise” within the data due to associations with retinal vascular changes and other risk factors. In particular, older age is associated with reduced vessel calibre^39^ in contrast to the increased venular calibre reported previously in association with DN. Older age is also strongly associated with a reduction in eGFR resulting in increased prevalence of CKD in older populations which may be sufficient to obscure any changes in retinal vascular morphology^40^. Although most studies adjust for the effects of age, the potential for confounding in cross-study comparisons, exists. Furthermore, although we adjusted for glycated haemoglobin, the potential confounding of insulin control may also influence variation in RVP between type 1 and type 2 diabetes.

Previously reported associations between RVP and renal outcomes in type 2 diabetes have not always been consistent. Data from the Wisconsin Epidemiological study of Diabetic Retinopathy (WESDR) reported an association between wider venular calibre and DN incidence over 14 years of follow-up^41^. In contrast, our data failed to support the findings from WESDR, but do support other cross sectional studies in type 2 diabetes^42^, and prospective data with similar 2-year follow-up duration^43^ suggesting RVP may not predict change in eGFR over a short time period. Inclusion of proteinuria or albuminuria measures with eGFR may prove more informative, given previous reported associations with RVP in both type 1 and type 2 diabetes^33–37,41^.

The majority of previous investigations have been limited to the analysis of vessel calibre, with only a single study considering fractal dimensions with regard to albuminuria in a young type 1 diabetes population^37^. We report novel findings for measures of vascular spread (retinal vessel fractal dimension), tortuosity, and branching patterns in type 2 diabetes. Fractal dimension and tortuosity were not associated with eGFR decline over the 3-year time-period in this type 2 diabetes cohort.

This study had several strengths. The prospective design allowed the predictive capacity of RVP to be examined over a 2–4 year period. Participant recruitment was clinically driven and electronic data record linkage provided an extensive range of variables on most participants. Our study provided novel data on the predictive utility of RVP and eGFR in type 2 diabetes and included wide range RVP previously under-reported in this context (i.e. fractal dimension, tortuosity, and number of first arteriolar branches). RVP were found to have no predictive value for 3-year change in eGFR in type 2 diabetes in our cohort with a mean age of 63 years. Our cohort originated from a population of type 2 diabetes from Tayside, Scotland, with healthcare record linkage which reduced the likelihood of bias. As a result, the findings are likely to be generalisable to other type 2 diabetes populations. The population was however almost entirely white and therefore, this sample is likely to be most closely generalisable to other predominantly white populations with type 2 diabetes of a similar age, given known associations of diabetes and renal disease with ethnicity. There is also evidence to suggest ethnic differences in retinal microvascular parameters^20^, perhaps in part due to factors associated with iris colour, retinal pigmentation and/ or underlying genetic influences^44^.

The limitations of this study include the case-control design which increased the likelihood of regression toward the mean, although the risk of regression toward the mean was reduced through the use of median eGFR values calculated from all available measurements recorded within 6 months either side of the date of each retinal photograph. The definition of progressors as those with eGFR < 60 ml/min/1.73 m^2^ at follow-up, or a reduction in eGFR of at least 15% between baseline and follow up, risked the inclusion of participants with limited reduction in renal function (e.g. eGFR 60 ml/min/1.73 m^2^ at baseline, and 59 ml/min/1.73 m^2^ at follow-up). This proved not to be an issue as only two participants had eGFR < 60 at follow-up combined with reductions in eGFR smaller than −15%, and each of these had reductions in eGFR from baseline exceeding 14% (data not shown).

Another limitation was the 3-year duration between baseline and follow-up measures, which may have been insufficient to detect associations between RVP and change in eGFR. As both retinal calibre and eGFR decline over time, the age (mean age 63 years, SD 7.6) of the population may also have limited the sensitivity to detect such associations with RVP. A study with longer follow-up, including participants entering at a younger age may be required to detect such associations.

A more comprehensive assessment of renal function (such as albumin / creatinine ratio (ACR)) and/or appropriate GFR estimating equations in ‘at risk’ individuals may have improved the sensitivity of our approach and comparability with other studies. Unfortunately, proteinuria/ACR data was not available for the earlier phase of GoDARTS recruitment. Although, HbA_1C_ was included in regression models, duration of diabetes was not, and may have a confounding influence. The suitability and size of our sample may also have limited our capacity to detect meaningful associations. Inclusion of additional individuals with baseline eGFR at the lower end of CKD stage 2 (60–70 ml/min/1.73 m^2^) may have provided more meaningful clinical significance as they transition from CKD stage 2 to stage 3.

The algorithms used by the retinal vessel measurement platform have been validated against the “gold standard” method of manual vessel tracing^45,46^. However, establishing the ground truth for validation of retinal vessel measurements is challenging because of the time intensive nature of the manual work and relatively poor inter-grader reliability that arises from manual assessment through expert disagreement on challenging issues related to defining vessel boundaries^47,48^. In contrast, the semi-automatic vessel assessment platform used in this study shows excellent inter-operator reliability^49^. Nevertheless, results may vary from associations based on manually assessed retinal vascular parameters. Furthermore, measures of vascular geometry in other locations of the retina, such as the macula, may be of particular interest. However, summary measures of vessel calibre within an annulus encircling the optic disc avoid problems created by variations in branching patterns between individuals, by including all of the largest microvessels of the eye^50^.

The results of this study suggest that retinal vascular calibre, fractal dimension, tortuosity, and number of first vascular branches surrounding the optic disc are not predictive of eGFR decline over a 3 year follow-up in this white population with type 2 diabetes.

## Methods

A nested longitudinal case-control design was undertaken in participants (n = 1072) from the Genetics of Diabetes Audit and Research in Tayside Scotland (GoDARTS) study cohort (ClinicalTrials.gov Identifier: NCT02783469). The GoDARTS cohort comprises 9,439 participants with type 2 diabetes and 8,187 individuals with similar demographics but without type 2 diabetes at the time of recruitment. The study was granted approval from the Tayside

Research Ethics Committees in Scotland and was carried out in accordance with the Declaration of Helsinki, and has been described elsewhere^51^. Briefly, participants in GoDARTS were identified from a central database of all patients registered with a general practitioner from the Tayside region of Scotland. Diagnosis of type 2 diabetes was made by physicians and participants provided informed consent and agreed to electronic healthcare record linkage. All electronic medical record data was processed and provided in an anonymised form for research through robust information governance procedures approved by local NHS Caldicott Guardians through the Health Informatics Centre at the University of Dundee.

**Figure 1:**
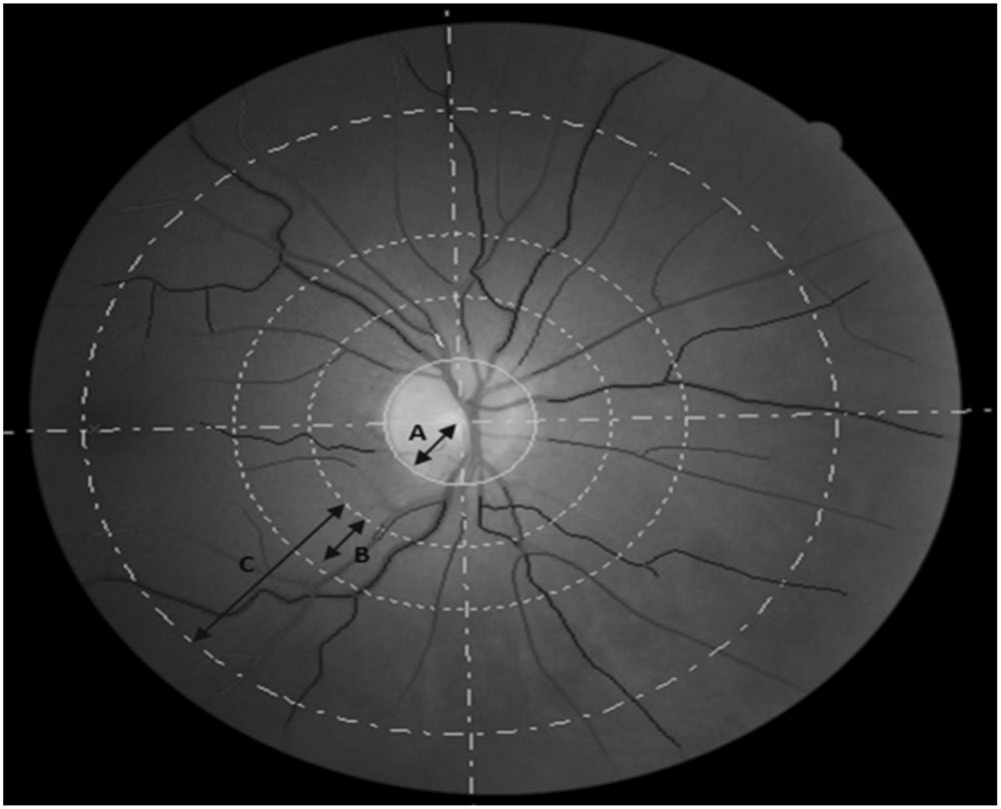
Retinal fundus photograph indicating vessels and zones of measurement. Figure 1 shows a retinal image centred on the optic disc. Line R indicates the optic disc radius, line B indicates the annulus 1 to 1.5 optic disc diameters from the centre of the optic disc (Zone B); line C indicates the annulus 1 to 2.5 disc diameters from the centre of the optic disc (Zone C). Zone B is the area of measurement for central retinal arteriolar equivalent and central retinal venular equivalent. Zone C is the area of measurement for fractal dimension. The dark and light lines in this greyscale reproduction indicate arterioles (light) and venules (dark) recognised by VAMPIRE vessel assessment software and corrected by a trained operator.

For inclusion in the present study, GoDARTS participants had to meet the following inclusion criteria: presence of type 2 diabetes, eGFR >60 ml/min/1.73 m^2^ at baseline, available digital retinal fundus images of sufficient quality for analysis at two time points (2–4 years apart) with corresponding clinical serum creatinine measurements within 6 months of each retinal image. Serum creatinine measurements were obtained from centralised Blood Sciences Laboratory records and retinal fundus images were obtained through routine diabetic retinopathy screening^52^. The earliest available digital fundus image of suitable quality for analysis was selected for the right eye with a follow-up image captured 2–4 years later from the same eye. Retinal fundus images were analysed using semi-automated software, Vessel Assessment and Measurement Platform for Images of the REtina (VAMPIRE; VAMPIRE group, University of Dundee, Dundee, Scotland) version 3.1^53,54^, by trained graders blinded to participant data. VAMPIRE 3.1 measures RVP within predefined annular zones: CRAE, CRVE, arteriovenous ratio (AVR), number of first vessel branches within a pre-defined zone C, fractal dimension and vessel tortuosity (Fig. 1). Intragrader reliability of retinal vascular measurements was measured using the intraclass correlation coefficient, assessed in four sessions of 20 retinal images at regular intervals over the course of the measurement period. Mean intraclass correlation coefficient for these sessions was calculated as 0.936 for CRAE and 0.950 for CRVE, respectively, indicating excellent operator alignment. Diabetic retinopathy status (presence/absence) was obtained from medical records.

The median serum creatinine values, HbA_1_c, SBP and DBP were calculated from all available measurements recorded within 6 months either side of the date of each retinal photograph. eGFR values were calculated from median serum creatinine values using the Chronic Kidney Disease Epidemiology Collaboration (CKD-EPI) equation^55^.

Participants were divided into two groups based on change in eGFR between both time points. Group 1, “non-progressors”, included participants with stable renal function or a reduction in eGFR <10% between both time points. Group 2, “progressors”, included participants with an eGFR of <60 ml/min/1.73 m^2^ at follow-up or a reduction in eGFR of at least 15% between baseline and follow-up.

The datasets generated during and/or analysed during the current study are available from the corresponding author on reasonable request.

Statistical analyses were performed using IBM SPSS v24 (Chicago, Illinois, USA). Participants with missing data were excluded from the analyses. Continuous variables were reported using means and SDs. Categorical variables were reported as percentages. Between and within-group comparisons were made using t-tests (two-sided significance reported) for continuous variables, and Chi-squared tests for proportions. The relationship between RVP and eGFR was assessed using logistic and linear regression models. Logistic regression models were used to test for association between baseline RVP and progression of renal disease. The independent variables were RVP and progressor/non-progressor designation as a binary dependent variable. Logistic regression models were carried out unadjusted, and adjusted for important known confounding variables; age, gender, baseline SBP, and baseline HbA_1_c. Additional models were used to further adjust for covariates related to RVP; diabetic retinopathy and fellow vessel calibre (for models including retinal vascular calibre as the independent variable). Associations involving continuous outcome variables were assessed using multiple linear regression. Linear regression models were used to test for cross-sectional association between renal function and RVP at follow-up. The independent variables were RVP, the dependent variable was eGFR. Linear regression models controlled for age, gender, follow-up SBP and HbA_1_c. Additional models adjusted for diabetic retinopathy and fellow vessel calibre (where retinal vascular calibre was the independent variable). Tortuosity variables were log transformed before linear regression to produce normal distributions and to conform to the assumptions of the analysis. All significance values reported are two-sided.
